# Simulation and Modelling of Hydrogen Production from Waste Plastics: Technoeconomic Analysis

**DOI:** 10.3390/polym14102056

**Published:** 2022-05-18

**Authors:** Ali A. Al-Qadri, Usama Ahmed, Abdul Gani Abdul Jameel, Umer Zahid, Muhammad Usman, Nabeel Ahmad

**Affiliations:** 1 Chemical Engineering Department, King Fahd University of Petroleum and Minerals, Dhahran 31261, Saudi Arabia; g201472160@kfupm.edu.sa (A.A.A.-Q.); a.abduljameel@kfupm.edu.sa (A.G.A.J.); uzahid@kfupm.edu.sa (U.Z.); 2 Interdisciplinary Research Center for Hydrogen and Energy Storage, King Fahd University of Petroleum & Minerals, Dhahran 31261, Saudi Arabia; muhammadu@kfupm.edu.sa; 3 Center for Refining & Advanced Chemicals, King Fahd University of Petroleum and Minerals, Dhahran 31261, Saudi Arabia; 4 Interdisciplinary Research Center for Membranes & Water Security, King Fahd University of Petroleum and Minerals, Dhahran 31261, Saudi Arabia; 5 Department of Chemical Engineering, COMSATS University Islamabad, Lahore Campus, Islamabad 54000, Pakistan; nabeelahmad@cuilahore.edu.pk

**Keywords:** gasification, reforming, plastic waste, H_2_ production, CO_2_ emissions

## Abstract

The global energy demand is expected to increase by 30% within the next two decades. Plastic thermochemical recycling is a potential alternative to meet this tremendous demand because of its availability and high heating value. Polypropylene (PP) and polyethylene (PE) are considered in this study because of their substantial worldwide availability in the category of plastic wastes. Two cases were modeled to produce hydrogen from the waste plastics using Aspen Plus^®^. Case 1 is the base design containing three main processes (plastic gasification, syngas conversion, and acid gas removal), where the results were validated with the literature. On the other hand, case 2 integrates the plastic gasification with steam methane reforming (SMR) to enhance the overall hydrogen production. The two cases were then analyzed in terms of syngas heating values, hydrogen production rates, energy efficiency, greenhouse gas emissions, and process economics. The results reveal that case 2 produces 5.6% more hydrogen than case 1. The overall process efficiency was enhanced by 4.13%. Case 2 reduces the CO_2_ specific emissions by 4.0% and lowers the hydrogen production cost by 29%. This substantial reduction in the H_2_ production cost confirms the dominance of the integrated model over the standalone plastic gasification model.

## 1. Introduction

Globally, 9% of plastics out of 6.3 billion tons have been recycled between 1950 and 2018. Additionally, 12% have been burnt [1]. However, the remaining 79% of plastics promote severely harmful pollutants. Those pollutants have different forms such as furans, dioxins, and mercury. The pollutants are highly hazardous, negatively affecting the environment and marine organisms [2]. Moreover, 4–12 million tons of plastics are annually thrown into the ocean [3]. Many countries are encouraging and legislating laws to minimize plastic usage, followed by recycling the plastics [4]. The efficient recycling of plastics to valuable products is essential to save the environment and utilize the energy from these huge amounts of waste. Several studies have confirmed the feasibility of plastic recycling [5,6].

The recycling process encompasses four main steps: collection, separation, manufacture, and marketing [7]. The most convenient technique is thermochemical recycling because it converts the plastics into synthesis gas, which could be used in synthesizing several valuable chemicals [8,9].

Gasification is a process that produces synthesis gas (CO_2_, CO, H_2_, CH_4_, etc.) from carbon-based materials such as fossil fuels, and biomass [10,11,12,13,14]. The syngas can then be used to produce several fuels and chemicals [15]. The gasification process is usually promoted through a high-temperature reaction (>700 °C) using oxygen or steam as an auxiliary component (air gasification or steam gasification) [16,17]. Steam gasification, air gasification, co-gasification, pyrolysis, and plasma gasification are types of thermal recycling for plastics or any carbon-based feedstock [18]. Pyrolysis is a dry heating of the feed in the absence of air [19]. The pyrolysis produces syngas that is completely free of tar [20]; however, the hydrogen to carbon monoxide ratio is not high. Sometimes, it is considered as the first step in the gasification process because it maximizes the conversion of volatile materials (high carbon chain) to relatively low carbon hydrocarbons (<C25) [21]. Another process is co-gasification, which mixes two carbonaceous feedstocks such as plastic with coal or biomass to enhance the gas yield and suppress char formation [22]. However, this process increases the tar formation [23]. Air gasification produces less tar; nevertheless, it produces a lower hydrogen to carbon monoxide ratio [24]. Pure oxygen gasification is a very efficient process; however, the production of oxygen from air is highly expensive [25]. To effectively produce syngas with a high hydrogen to carbon (HCR) ratio in a quite simple process, the steam gasification of plastics is the optimal choice [26]. It is quite simple, and it produces a higher hydrogen to carbon monoxide ratio.

The production of syngas facilitates the production of essential chemicals and fuels, such as hydrogen, methanol, ethanol, DME, LPG, olefins, and gasoline [27,28,29]. Modeling the whole journey of plastics to clean fuels under several operational conditions is essential to support industrial applications, and to maximize the clean fuel production from a heterogeneous plastic mixture [30]. Antzela and Ioanna [31] conducted a pilot plant study on the techno economic evaluation of the conversion of plastics into heavy oil through pyrolysis using Aspen HYSYS. The production cost of the heavy oil was 0.87 EUR /kg, which is 58% higher than the market price. They suggested a more sophisticated study for large-scale data. Deng et al. [32] modeled the municipal solid plastic (MSW) to syngas using a combination of two technologies: pyrolysis (RYield + RGibbs), and gasification (RGibbs). The results show good agreement with the experimental data, where the temperature of 750 °C is considered the optimal gasification temperature, with a steam/plastic ratio of 0.4. Furthermore, they economically recommended the use of flue gas and steam as gasifying agents. Another study by Pravin et.al [33] accomplished the conversion of PE (polyethylene) to syngas through pyrolysis then gasification using Aspen plus. The results were not validated by the experiment due to the lack of resources; however, they claimed that the most convenient temperature, and equivalence ratio for the pyrolysis unit were 0.4–0.6, and 500–750 °C, respectively The catalytic approach has advantages over the thermal one in terms of reducing the sulfur content when special catalysts are used (i.e., CaS, and MgS) [34,35,36]. Several studies have been performed on the conversion of waste plastics to hydrogen along with other feedstocks [37,38]. The development of catalysts for plastic gasification in a cost-effective manner is still under research; therefore, the thermal gasification technique is considered, which is a well established process with fewer operational issues.

Fivga and Dimitriou [39] studied and analyzed the conversion of waste plastics to heavy fuel. They used a mixture of PE, PP, and PS as a feedstock at 530 °C, and 1 atm. They modeled their work using Aspen HYSYS based on the ultimate analysis of the plastics. The product of their pyrolysis reactor was basically n-C30, n-C25, n-C18, n-C14, n-octane, ethane, and a small proportion of gases. The remaining solids and gases were separated, then pyrolyzed liquid fuel was collected. They validated their results with plant data, and they performed cost analysis. Generally, the work is promising and has the idea of using plastic waste to generate liquid heavy fuel. Another study on plastic waste conversion to fuel was conducted by Emad and Vahid [40], which was basically on the production of hydrogen via the co-gasification of a mixture of asphaltene and plastics using Aspen Plus. They decomposed the feed on a pyrolysis reactor (RYield), and then they used an RGibbs reactor followed by CSTR to produce syngas. They studied some factors influencing the hydrogen production rate, namely, asphaltene to plastic ratio (A/P), equivalence ratio (ER), and steam to feed ratio (S/F). They found that A/P and steam to feed (S/F) have a positive impact on carbon conversion efficiency (CCE). The study provided the excellent idea of producing hydrogen from a co-gasification mixture. However, they did not produce pure hydrogen; it was a synthesis gas mixed with acid gases that should be removed. Additionally, they need to implement WGS to maximize hydrogen production and to suppress the carbon monoxide in the product.

There are limited studies on the production of hydrogen from plastic wastes. Therefore, investigating the hydrogen production from different feedstocks (i.e., coal or biomass) will assist hydrogen production from plastics. A study was performed by Noussa et al. [41] on the techno economic evaluation of producing H_2_ from biomass. They investigated different gasifier agents and several types of feedstocks. They found that steam as gasification agent was better than other agents. Namioka et al. [42] studied the production of H_2_-rich synthesis gas using pyrolysis, then low-temperature steam gasification. The study was focused on polystyrene (PS), and polyethylene (PE) as a feedstock. They performed the pyrolysis and steam reforming at 673 and 903 K, respectively. Ruthenium was used as a pyrolysis catalyst, and it enhanced the process performance. The study recommended combining the thermal and catalytic process. Similarly, Chaia et al. [43] studied the conversion of plastics to hydrogen using a combination of co-pyrolysis and gasification processes. Ni-CaO-C was tested as a novel catalyst to promote H_2_ production. They claimed a hydrogen production efficiency of 87.7 mole %, controlling the greenhouse gas emissions. Consequently, the conversion of waste plastics into hydrogen is a practical process, proved theoretically and experimentally. Thus, the current study will focus on using a thermochemical approach based on steam gasification to convert plastics into hydrogen fuel [42].

## 2. System and Analysis Framework

### 2.1. Modelling and Simulation Approach

In this study, polyethylene (PE) and polypropylene (PP) were selected due to their availability and their higher heating value [44]. Aspen Plus^®^ software V-12 was used as a simulation tool, selecting Peng Roberson (PR) as an appropriate property package. It is generally recommended for oil and gas systems [45,46]. There are several classifications of plastics in terms of composition. The approximate and ultimate analyses of the plastic feedstock are provided in Table 1. To specify the plastic heating value, the HCOALGEN model was selected. Prior to generating syngas, the RYield reactor was simulated to break down the solid feedstock, and then the outlet mixture was fed to the gasifier (i.e., RGibbs reactor). The products were mainly syngas containing CO, H_2_, and CO_2_. The RGibbs reactor operated at a high temperature (i.e., 900 °C). The outlet syngas was introduced to water gas shift (WGS) to convert CO to hydrogen via the WGS reaction in two REquil reactors. The reactions are given in Table A1.

The process operational conditions were set based on previous studies with several assumptions. Table 2 illustrates the major assumptions made in the whole process. The primer design of the model was based on a study conducted by Dang et al. (2019) [47].

Standalone models for polyethylene and polypropylene were developed and validated with the literature results based on the experiments [26,48]. For the purpose of validation, the same process conditions used in the simulation model were kept in the experimental setup. Table 3 represents the comparison between the experimental and the simulation results for plastic gasification. The simulation results are in good agreement with the experimental results and the simulation models can be used with confidence for hydrogen production.

### 2.2. Development and Validation of Case Studies

#### 2.2.1. Case 1 (Base Case)

**Figure 1:**
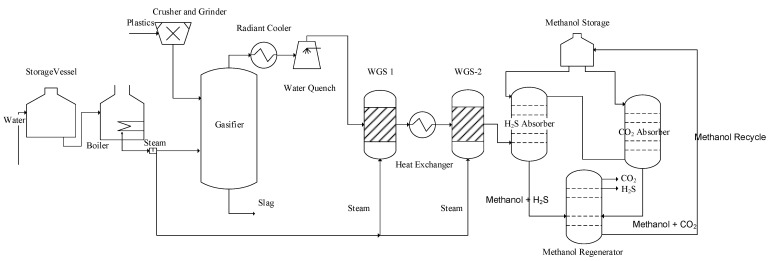
Hydrogen production from waste plastics: PE and PP (case 1).

Figure 1 represents the general process flow diagram of case 1. The mixture of PE and PP in the equal weight ratio of 50:50 was crushed and fed to the steam gasification unit to generate the syngas. The solid plastics were first decomposed in the decomposer (RYield) and then fed to the gasification unit to produce the syngas at a temperature of 900 °C, where a hydrogen to CO ratio of 1.86 was achieved. Then, the syngas was quenched to sustain WGS reactions. The outlet stream from WGS reactors mainly included hydrogen, CO_2_, and some traces of H_2_S, where the ratio of H_2_/CO_2_ was obtained as 2.86. Methanol was selected as an absorbent in the AGR unit to remove hydrogen sulfide and carbon dioxide, where the methanol was recovered in the H_2_S and CO_2_ regenerator columns.

#### 2.2.2. Case 2 (Alternative Case)

**Figure 2:**
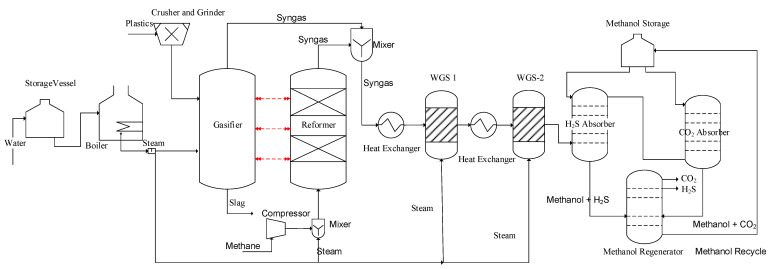
Hydrogen production from plastic waste gasification integrated with reforming (case 2).

Case 2 is similar to case 1 in terms of the gasification process; however, case 2 contains an additional process unit. The alternative case (case 2) represents the integration of the steam methane reforming (SMR) model with the plastic gasification model to utilize the gasifier’s heat energy in the reforming unit, making it different from case 1. The process base flow diagram is provided in Figure 2. The steam to methane molar ratio was set as 1.50 and the inlet temperature was selected as ~900 °C. The process reactions are provided in Table A1. The SMR results were also validated with the literature in terms of hydrogen to carbon monoxide ratio, which was found to be around 3.0 [49]. The syngas mixture obtained from SMR and gasification was mixed and introduced to WGS with the same conditions applied in the base case design, and were also used in case 2. Finally, the acid gas removal unit was used to remove the CO_2_ and H_2_S from the gas streams to obtain pure hydrogen.

### 2.3. Governing Equations for Technical Analysis

Table A2 represents some of the equations used in this study for technical and economical comparison between the two cases. The lower heating value (LHV) was calculated based on the mole fraction of hydrogen and carbon monoxide [47]. HPF is an indicator that represents the hydrogen per total feed in terms of mass basis. The hydrogen thermal energy was calculated from the lower hydrogen heating value considering the flow rate of hydrogen. The specific carbon dioxide emissions and process efficiency indicators were also used for the comparison between two cases [50]. The total investment cost (TIC) represents the capital cost with respect to the hydrogen production rate. The capital investment for each unit was calculated from previous similar studies considering the Chemical Engineering Plant Cost Index (CEPCI). To assess the operating expenditures, total manufacturing cost was computed as the sum of maintenance, administrative, labor, support, and overhead costs. The utility and labor costs were calculated based on Donald E. Garrett [51]. The levelized hydrogen production cost was estimated for 30 years considering the total hydrogen produced in a lifetime and the expense incurred.

## 3. Results and Discussion

Case 1 and case 2 are compared in terms of hydrogen production rates, syngas heating values, hydrogen purity, carbon emissions, production cost and the process feasibility. The equation given in Table A2 was used for the comparative analysis.

### 3.1. Technical Analysis

#### 3.1.1. Syngas Production and Analysis

The feedstock mainly consists of polyethylene and polypropylene. The feedstock is fed with a mass ratio of 1:1. The total plastic flow rate is considered as 100 kg/h, where the steam to plastic ratio is maintained as 1.25:1. The natural gas flow rate in case 2 is taken as 42 kg/h, with a steam to natural gas ratio of 1.6:1. It can be seen from the results that the molar ratio of H_2_/CO for case 1 and case 2 is 1.86 and 2.23, respectively. The hydrogen to carbon monoxide ratio was enhanced in the second case by 62% compared with case 1. The carbon dioxide emission after WGS reactors was lower in case 2 than case 1 by 11%. Overall, the results reveal that case 2 is more efficient than the base case in terms of syngas heating value and carbon dioxide emissions. Table 4 provides the operational conditions, and the stream flow rates at the outlet of all the essential units.

**Figure 3:**
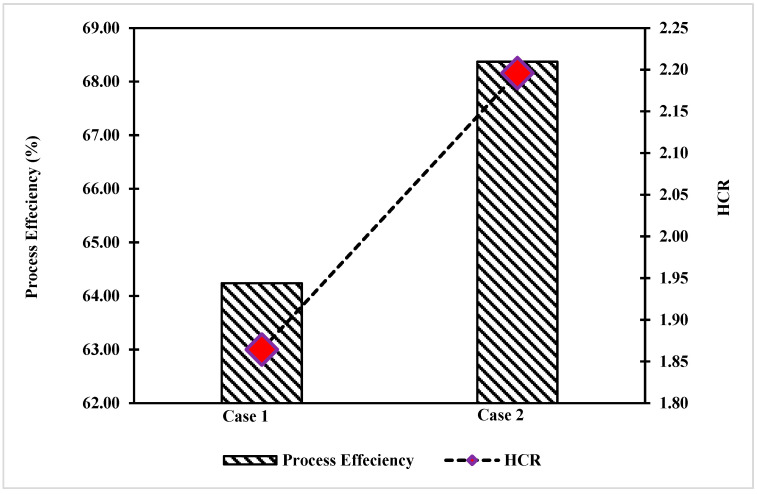
Comparison of process efficiency and hydrogen to carbon monoxide ratio for case 1 and case 2.

To determine the efficiency of the process, syngas composition is a key parameter for such evaluation. The HCR at the inlet of WGS was evaluated for case 1 and case 2, as given in Figure 3. It was found that the H_2_/CO was higher in case 2 than case 1, indicating a higher heating value for the integrated case.

#### 3.1.2. Overall Process Performance

The overall process efficiency was investigated for the two cases by considering several essential parameters, as given in Table 5. The first parameter is the hydrogen purity at the outlet of the acid gas removal unit, which is greater in the second case by 0.15% compared with the first case. The process’s overall efficiency in feedstock conversion was calculated and is represented by HPF, which was found to be greater in case 2 by 5.6%. Additionally, the two cases were evaluated in terms of heating value (i.e., lower, and higher heating values). The results show that the second case had a lower heating value (LHV) and higher heating value (HHV) than case 1 by 5.7%, and 5.0%, respectively.

The integrated process produced more hydrogen than the classical one because the SMR unit had higher hydrogen production. The overall energy process efficiency was calculated and studied, and it was higher in case 2 than case 1 by 4.13%. Thus, case 2 is more efficient than case 1 in terms of syngas heating value. However, economic analysis will be performed to confirm the final preference for the alternative design.

#### 3.1.3. CO_2_ Specific Emissions

**Figure 4:**
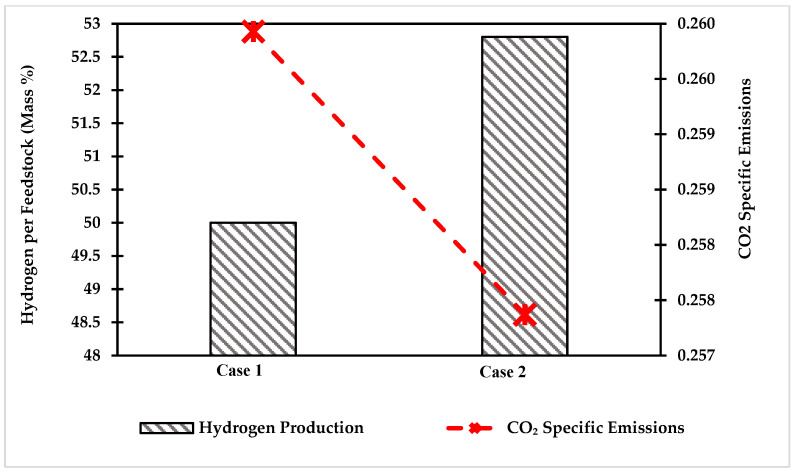
Comparison of hydrogen production and specific CO_2_ emissions for case 1 and case 2.

Another essential factor in the investigation and comparison of the two designed cases is the specific emission of carbon dioxide. Case 2 showed lower carbon emissions than case 1 by 1.2%. A study conducted by Usman et al. [37], about the conversion of coal to hydrogen, found that the specific CO_2_ emissions were in the range of 0.70 on a mass basis. Figure 4 shows the specific carbon dioxide emissions for each case along with HPE (hydrogen per total feed; mass ratio). The HPF for case 2 is higher than case 1, with lower carbon emissions. The results show that the alternative case produces a higher amount of hydrogen with minimal carbon dioxide emissions.

#### 3.1.4. Sensitivity Analysis on the Gasifier

To optimize the design parameters, sensitivity analysis was performed mainly on the gasification unit. The main factors affecting the process performance are the gasification temperature, pressure, steam to feed ratio, and PE and PP blending ratio.

##### 3.1.4.1. Steam to Feed Ratio Effect on Syngas Composition

**Figure 5:**
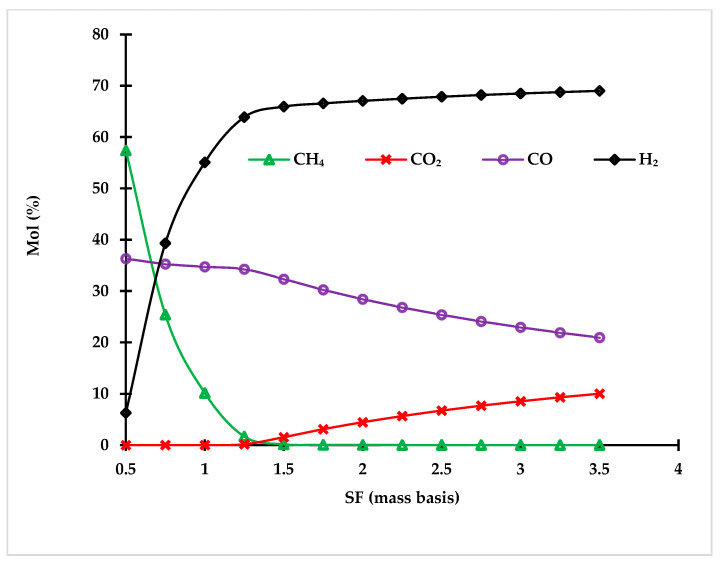
Effect of steam to plastic ratio on syngas composition.

The steam to feed ratio has a strong effect on the gasification process because it significantly controls the outlet syngas composition. Figure 5 represents the sensitivity analysis of the steam gasification unit when investigating the impact of the steam to plastic ratio (S/P or S/F) on syngas composition. Increasing the steam to plastic ratio decreases the CO production rate; however, it dramatically enhances the hydrogen production. CH_4_ is suppressed when S/P increases. The optimal steam to feed ratio at 900 °C, as deduced from the figure, is 1.25, because any further increase had a negligible impact on syngas LHV. The analysis was performed on the blend of PE and PP based on equal weight. The results show that increasing the steam to plastic ratio has a positive impact on enhancing the syngas heating value; however, going beyond a steam to plastic (S/P) ratio of 1.25 decreases the heating values of syngas by producing more CO_2_.

##### 3.1.4.2. Temperature Effect on Syngas Composition

**Figure 6:**
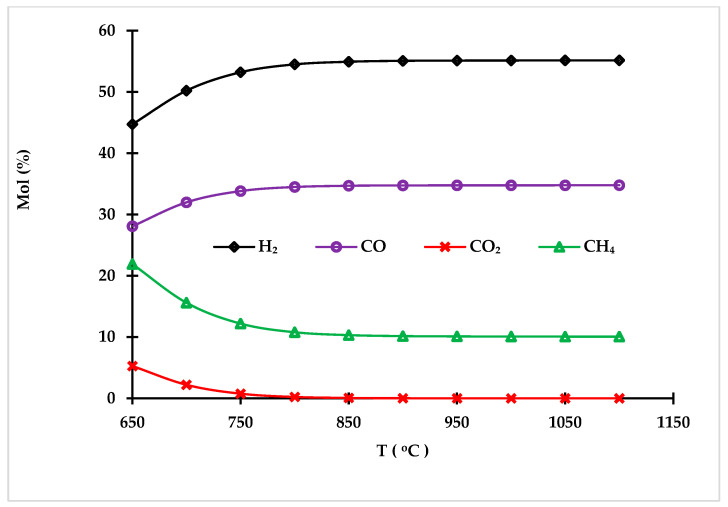
Effect of gasification temperature on syngas composition.

A lower gasification temperature promotes higher carbon dioxide production due to the Boudouard reaction, which is the reaction of CO_2_ with carbon to produce CO. It is an endothermic reaction; therefore, as the temperature increases, less carbon dioxide is produced [52]. Increasing the temperature has a positive effect on the heating value of syngas. Increasing the temperature up to 900 °C has a positive effect on the heating values and produces more hydrogen. Figure 6 shows the impact of gasifier temperature on the gasification process at an S/F of 1.0, and a PE/PP of 1:1. Therefore, the gasification temperature of 900 °C was considered for both cases to achieve maximum hydrogen production and a high heating value of syngas.

### 3.2. Economic Analysis

The economic analysis is essential to comprehend the process’s feasibility and promotability and to precisely compare the two designs. The order-of-magnitude cost analysis has been used to determine the capital cost [53]. Several assumptions were considered to perform the economic analysis. Table A3 represents the assumptions to accomplish the economic analysis [50]. The waste plastic price was ignored in both cases, and the plant life was considered for 30 years using an exponent factor (x) of 0.6 for consistent analysis. Three shifts per day were considered with a stream factor of 0.95 to estimate the operational expenses. The working capital, land and salvage were each taken to be 10% from the fixed capital investment. The offsite unit and utilities were taken to be 25% of the equipment and installation cost. The contingency and permitting costs were chosen to be 15% and 5% of the equipment and installation cost, correspondingly. The discount rate and taxation rate were assumed to be 8% and 15%, respectively, in both cases.

#### 3.2.1. Estimation of CAPEX and OPEX

The capital expenditure (CAPEX) and the operational expenditure (OPEX) are the two important parts of a project’s economic evaluation. The total investment is impacted by various variables such as the capacity of the plant, raw materials, operational time, and the process efficiency. The fixed CAPEX predominantly comprises the equipment and plant facilities costs. This study used the power law to estimate the CAPEX with a capacity factor (x) of 0.6, as mentioned in Table A3. The power law uses the concept of Chemical Engineering Plant Cost Index (CEPCI). Table 6 represents the CAPEX cost summary, calculating some of the important parameters such as total investment cost (TIC). The total investment cost for the two cases has a huge difference due to the variation in the process configuration and the type and capacity of the plant. The total investment cost (FCI) in terms of MMEUR for case 1 and case 2 was calculated as 3.79 and 4.46, respectively. The FCI for the alternative case was higher than that of the base case because case 2 has an SMR process with an additional feedstock (i.e., natural gas). The TIC represents the total investment cost per hydrogen production rate in tons. The TIC for the alternative case was higher than the base case by 23%, indicating the cost-effectiveness of the alternative case in terms of capital expenditure while considering the production rate of hydrogen.

On the other hand, the operational cost of the project is represented by OPEX, which is classified into two different categories. The two categories are the fixed OPEX and variable OPEX. The fixed OPEX involves the maintenance, labor, and administrative costs, where the variable OPEX encompasses the fuel, catalysts, waste disposal, and boiler feed water costs. Table 6 shows the OPEX summary for the two designed cases. The total OPEX in MMEUR/year for case 1 and case 2 is calculated as 1.39 and 1.47, respectively. The total operational expenditures are higher in case 2 than case 1; however, when the production rate of the fuel is considered, case 2 shows a 30% reduction in the operational cost per ton of hydrogen production. The revenue calculated for both cases revealed that case 2 offers 51% higher revenue than case 1.

#### 3.2.2. Cash Flow and Hydrogen Cost Analysis

**Figure 7:**
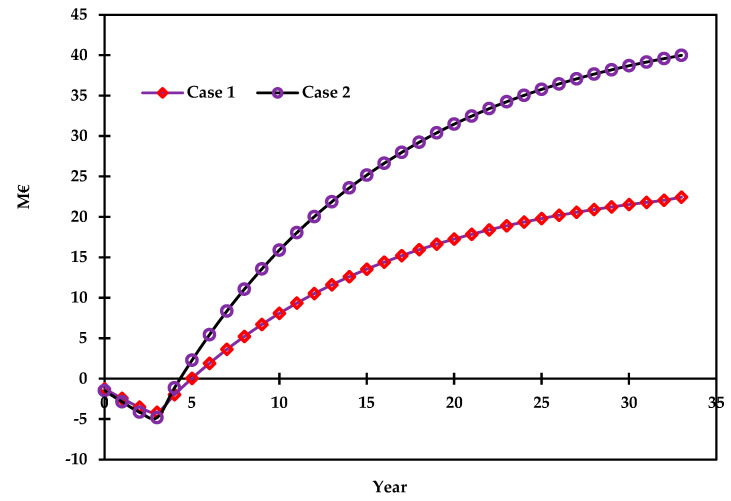
Cash flow diagram for case 1 and case 2.

The purpose of this section is to provide the cash flow diagram and to compare the levelized hydrogen production rate with the literature. The TIC per ton of hydrogen was calculated as 76.53 MMEUR /ton and 59.25 MMEUR /ton for case 1 and case 2, respectively. Additionally, the levelized lifetime hydrogen production cost was calculated as 3.78 EUR /kg and 2.56 EUR /kg for case 1 and case 2, respectively. This indicates that case 2 produces hydrogen with 1.22 EUR less compared with case 1 for every kilogram of hydrogen produced. Figure 7 shows the cash flow diagram over the lifetime of the project for both cases [54]. The cash flow return on investment was higher for case 2 than the base case by 52%. Additionally, the net present value (NPV) was higher in the alternative case when compared with the base case by 78%. The present value ratio (PVR) for case 2 was found to be higher than case 1 by 45%. Overall, case 2 offered better process economics compared with case 1.

#### 3.2.3. Comparison of Hydrogen Cost with the Literature

**Figure 8:**
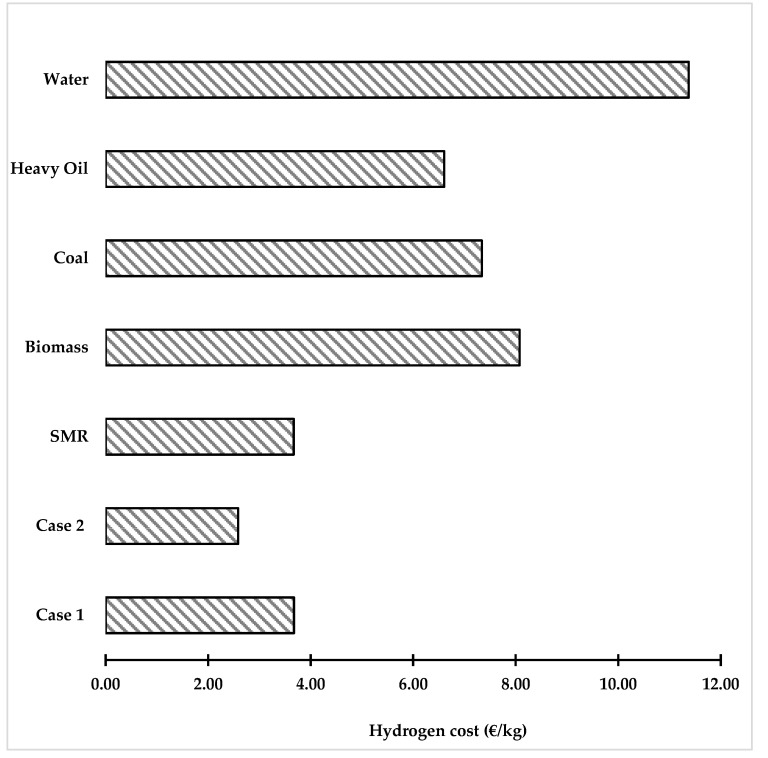
Comparison of hydrogen production costs from waste plastic with those of the literature.

Figure 8 compares the hydrogen cost obtained from our study with the literature considering different feedstocks [55,56]. The production of hydrogen via the solar and photovoltaic electrolysis of water has the highest hydrogen cost. It is considered as a green process; however, it consumes more energy. Biomass, coal, and heavy oil can produce hydrogen with a lower cost than the solar process. It was analyzed from the literature that the hydrogen production cost ranges from 5 to 8 EUR /kg [57] depending on the type of feedstock and the technology used for hydrogen production. From the comparative analysis, case 2 was found to be an attractive approach for hydrogen production with lower costs and shows potential to resolve the global plastic waste issue. The catalytic plastic gasification could also further reduce the hydrogen production cost because it is usually performed at a lower temperature [58]. Dan et al. [59] performed a study converting plastic wastes and biomass to hydrogen using Ni/γ-Al_2_O_3_ as a catalyst with a temperature of 800 °C. This might be a future direction in enhancing the conversion of waste plastic to hydrogen.

## 4. Conclusions

The study represents the hydrogen production from plastics (polyethylene and polypropylene) through two developed cases in Aspen Plus^®^. Case 1 is the thermochemical steam gasification process for converting waste plastics to hydrogen. On the other hand, case 2 represents a modified version of case 1 by integrating the steam gasification model with the steam reforming model to enhance the overall hydrogen production. The technical and economic analyses were performed for both cases to evaluate the feasibility of the process and are summarized as follows:

1. The H_2_/CO of the syngas for case 1 and case 2 is calculated as 1.86 and 2.23, respectively, whereas case 2 showed 19.78% higher values.
2. The hydrogen production rate per unit of feedstock for case 1 and case 2 is calculated as 50% and 52.2%, respectively.
3. The overall process efficiency for case 1 and case 2 is calculated as 64.24% and 68.37%, respectively, whereas case 2 shows 4.13% higher efficiency.
4. The TIC per ton of H_2_ calculated for case 1 and case 2 is 76 and 59 EUR/ton, whereas case 2 has the potential to increase the revenue by 51.7%.
5. Case 2 showed the potential to lower CO_2_ emissions by 1.0%.

## Figures and Tables

**Table 1:** Plastics and natural gas composition.

Plastic Composition Analysis		
Proximate Analysis (Weight %)		
	PE	PP
**Moisture**	0.02	0
**Ash**	0.15	0.7
**Volatile matter**	99.83	99.30
**Total**	100	100
**LHV (MJ/kg)**	38.04	44.70
**Ultimate analysis (weight %)**		
**Carbon**	85.81	86.23
**Hydrogen**	13.86	12.28
**Nitrogen**	0.12	0.62
**Sulfur**	0.06	0.17
**Ash**	0.15	0.7
**Total**	100	100
**Natural gas composition (mol %)**		
**CH_4_**	93.9	
**C_2_H_6_**	3.2	
**C_3_H_8_**	0.7	
**C_4_H_10_**	0.4	
**CO_2_**	1.0	
**N_2_**	0.8	
**Total**	100	
**LHV(MJ/kg)**	47.76	

**Table 2:** Design assumptions made for case 1 and case 2.

Equipment	Aspen Model	Assumption
**Plastic Flow Rate**	RYield/RGibbs	Plastics = 100 kg/h Entrained flow gasifier; steam:plastic = 1.25; Temperature = 900 °C; P = 1 atm
**Pre-reformer**	RStoic (reactor)	Heavier hydrocarbon hydrocracking
**Reformer**	RGibbs (reactor)	Temperature = 894.3 °C, pressure = 3 bar, Steam: NG = 1.6; nickel-based catalyst
**Water Gas Shift (WGS)**	REquil (reactor)	Two equilibrium reactors Steam:CO = 2:1 (molar basis)
**Acid Gas Removal (AGR)**	RadFrac and flash drums	Rectisol process; temperature = −30 °C, P = 1 bar CO_2_ removal = 99%; H_2_S removal = 10 ppm

**Table 3:** Polyethylene and polypropylene gasification validation.

**Validation of Polypropylene Gasification**			
**Component**	Reference Case	Base Case	Difference
**H_2_**	68.3	66.4	1.9
**CO**	26.1	27.5	−1.4
**CO_2_**	3.9	5.7	−1.8
**CH_4_**	1.3	0.3	1.0
**Others**	0.3	0.1	0.2
**Validation of Polyethylene Gasification**			
**Component**	Reference Case	Base Case	Difference
**H_2_**	68.6	67.4	1.2
**CO**	25.5	28.8	−3.3
**CO_2_**	1.1	3.7	−2.6
**CH_4_**	3.6	0.0	3.6
**Others**	1.2	0.0	1.2

**Table 4:** Flow rates and stream compositions at the exit of each unit.

	Plastics	Steam (Gasifier)	Gasifier	Reformer	Cooling and Syngas Mixing		WGS Unit		AGR Unit (H_2_ Storage)		CO_2_ Storage	
	Case 1 and 2	Case 1 and 2	Case 1 and 2	Case 2	Case 1	Case 2	Case 1	Case 2	Case 1	Case 2	Case 1	Case 2
**T (°C)**	300	300	900	894.3	220	220	10	10	25	25	25	25
**P (bar)**	1.013	1.013	1.013	3	1	1	1	1	1	1	1	1
**Mass Flow (kg/h)**	100	125	225	109	224.58	333.58	469.38	578.38	49.58	75.22	226.40	337.53
**Mole (%)**												
**H_2_**		-	0.636	0.683	0.636	0.653	0.579	0.654	0.976	0.978	0.0026	0.0026
**CO**		-	0.341	0.206	0.341	0.292	0.001	0.004	0.002	0.006	0.341	0.206
**CO_2_**		-	0.002	0.020	0.002	0.008	0.202	0.206	0.003	0.003	0.993	0.994
**H_2_O**		1	0.004	0.089	0.004	0.034	0.206	0.128	0	0	0	
**CH_4_**		-	0.017	0.001	0.017	0.011	0.010	0.008	0.016	0.011	0.0018	0.0012
**N_2_**		-	0.0008	0.0018	0.0008	0.0011	0.0004	0.0008	0.0007	0.0012	0.0008	0.0018
**H_2_S**		-	0.0002	-	0.0002	0.0001	0.0001	0.0001		-	0.0002	-
**CH_3_OH**		-	0.0000	-	0.0000		-		0.0018	0.0018	0.0000	-
**Molar H_2_/CO**	-	-	-	3.32	1.86	2.23	-	-	-	-	-	-
**Molar H_2_/CO_2_**	-	-	-	33.34	389.50	77.43	2.86	3.18	-	-	-	-

**Table 5:** Energy analysis.

Characteristic/Model Type	Case 1	Case 2
**Hydrogen per feedstock HPF (mass %)**	50	52.8
**Hydrogen purity (mole %)**	97.62	97.77
**Syngas gross heating value GHV (MJ/kg)**	26.18	27.67
**Syngas net heating value LHV (MJ/kg)**	23.55	24.73
**Feed stock energy (kWth)**	1198.61	1757.07
**Thermal energy of produced H_2_ (kWth)**	1385.25	2060.59
**Minimum hot utilities required (kW)**	757.06	1069.75
**Minimum cold utilities required (kW)**	200.80	187.06
**Total energy required after heat integration (kW)**	957.86	1256.81
**Process efficiency(ηnet) (%)**	64.24	68.37

**Table 6:** CAPEX and OPEX for case 1 and case 2.

**Capital Expenditure**		
**Equipment**	**Case 1 EUR (10^3^)**	**Case 2 EUR (10^3^)**
**Gasification price**	110	110
**Acid gas removal unit**	1339	1624
**Solid handling facility**	522	522
**Syngas processing unit**	646	690
**Reformer cost**	0	128
**Equipment and installation cost**	2617	3074
**Offsite unit and utilities**	654	768
**Contingency cost**	393	461
**Permitting**	131	154
**Total investment cost**	3795	4457
**TIC per ton of H_2_ MMEUR /ton**	76.53	59.25
**Operational expenditure**		
**Cost sector/designed case**	**Case 1 EUR (10^3^)/Year**	**Case 2 EUR (10^3^)/Year**
**Maintenance cost (2% of equipment and installed cost)**	52.3	61.5
**Labor cost**	459.4	472.9
**Administrative, support and overhead cost**	137.8	141.9
**Total fixed manufacturing cost**	649.6	676.2
**Natural gas**	0.0	16.5
**WGS catalyst**	16.6	18.0
**Reforming catalyst**	0.0	0.5
**Solvent**	39.0	57.8
**Waste disposal**	7.1	7.1
**Utility costs**	677.9	693.2
**Total OPEX/year**	1390.0	1469.3
**Total OPEX/ton H_2_**	3.4	2.3
**Revenue (MMEUR /year)**	4.804	7.289
**NPV**	22.450	39.978
**PVR**	6.401	9.288

## Data Availability

The data may be available upon request to the corresponding author.

## Appendix A

**Table A1:** Chemical reactions comprehended in the process.

**Gasification Reactor**	
$C_{\left(s\right)}+H_{2}O\;↔CO+H_{2}$	$\Delta H=+131\frac{MJ}{kmol}$
$C_{\left(s\right)}+CO_{2}\;↔2CO$	$\Delta H=+172\frac{MJ}{kmol}$
$C_{\left(s\right)}+2H_{2}\;↔\;CH_{4}$	$\Delta H=-74.8\frac{MJ}{kmol}$
$CO+H_{2}O↔CO_{2}+H_{2}$	$\Delta H=-41.2\frac{MJ}{kmol}$
$CH_{4}+H_{2}O↔CO+3H_{2}$	$\Delta H=+206\frac{MJ}{kmol}$
**Steam Methane Reforming Reactor**	
$3C_{2}H_{6}+H_{2}O\;\to 5\;CH_{4}+CO$	$\Delta H=+3.6460\frac{MJ}{kmol}$
$3C_{3}H_{8}+2H_{2}O\;\to 7\;CH_{4}+2\;CO$	$\Delta H=+16.607\frac{MJ}{kmol}$
$3C_{4}H_{10}+3\;H_{2}O\;\to 9\;CH_{4}+3\;CO$	$\Delta H=+41.116\;\frac{MJ}{kmol}$
$CH_{4}+2\;O_{2}\;\to \;CO_{2}+2\;H_{2}O$	$\Delta H=-802.54\frac{MJ}{kmol}$
$CH_{4}+H_{2}O\;\to \;CO+3\;H_{2}$	$\Delta H=+206.12\frac{MJ}{kmol}$
**Water Gas Shift Reactor**	
$CO+H_{2}O↔H_{2}+CO_{2}$	$\Delta H=-41\frac{MJ}{kmol}$

**Table A2:** Equations used for technical and economic appraisal.

Equations	**Eq. No.**
$LHV_{Syngas}=12.636\;y_{CO}+10.798\;y_{H_{2}}$	(A1)
$\mathrm{HPF}=\frac{\mathrm{Produced}\;H_{2}\;\left(\frac{\mathrm{kg}}{h}\right)}{\mathrm{Total}\;\mathrm{Feed}\;\left(\frac{\mathrm{kg}}{h}\right)}\times 100\%$	(A2)
$H_{2}\;\mathrm{Thermal}\;\mathrm{Energy}=H_{2}\mathrm{LHV}\;\left(\frac{\mathrm{kJ}}{\mathrm{kg}}\right)\times \mathrm{Produed}\;H_{2}\;\left(\frac{\mathrm{kg}}{h}\right);\mathrm{LHV}=100.539\frac{\mathrm{MJ}}{\mathrm{kg}}$	(A3)
Total Consumed Energy (kW)=Hot Utility (kW)+Cold Utility (kW)	(A4)
$CO_{2}\;\mathrm{specific}\;\mathrm{Emissions}=\frac{\mathrm{Uncaptured}\;CO_{2}\;\left(\frac{\mathrm{kmol}}{h}\right)}{H_{2}\;\mathrm{Production}\;\left(\frac{\mathrm{kmol}}{h}\right)}$	(A5)
$\mathrm{Process}\;\mathrm{Efficiency}\;\left(\eta_{\mathrm{net}}\right)=\frac{H_{2}\;\mathrm{Thermal}\;\mathrm{Energy}\;\left(\mathrm{kW}\right)}{\mathrm{Feed}\;\mathrm{Thermal}\;\mathrm{Energy}\;\left(\mathrm{kW}\right)\;+\;\mathrm{Energy}\;\mathrm{Consumed}\;\left(\mathrm{kW}\right)}\times 100\%$	(A6)
$\mathrm{Cos}t_{\mathrm{new}}=\mathrm{Cos}t_{\mathrm{old}}\times {\left(\frac{\mathrm{Capacit}y_{New}}{\mathrm{Capacit}y_{Old}}\right)}^{x}\times \frac{\mathrm{CEPC}I_{\mathrm{New}}}{\mathrm{CEPC}I_{\mathrm{Old}}}$	(A7)
Total Fixed Manufact Cost = Maintenance + Labor + Admin, support and overhead costs	(A8)
$N_{\mathrm{OL}}={\left(6.29+0.23\;N_{\mathrm{np}}\right)}^{0.5}$ $\;N_{OL}\;\mathrm{is}\;\mathrm{the}\;\mathrm{number}\;\mathrm{of}\;\mathrm{operators}\;\mathrm{per}\;\mathrm{shift}\;\mathrm{and}\;N_{np}\;\mathrm{is}\;\mathrm{nonparticulate}\;\mathrm{processing}\;\mathrm{steps}$	(A9)
$\mathrm{TIC}\;\mathrm{per}\;\mathrm{ton}\;\mathrm{of}\;H_{2}=\frac{\mathrm{Total}\;\mathrm{investment}\;\mathrm{cost}}{\mathrm{Hydrogen}\;\mathrm{generation}}$	(A10)
$\mathrm{Hydrogen}\;\mathrm{Cost}\;\left[\frac{EUR\;}{kg}\right]=\frac{\mathrm{Hydrogen}\;\mathrm{Life}\;\mathrm{Cost}\;\left(EUR\;\right)}{\mathrm{Hydrogen}\;\mathrm{Life}\;\mathrm{Production}\;\mathrm{Flow}\;\mathrm{Rate}\;\left(kg\right)}$	(A11)

**Table A3:** The assumptions for economic analysis.

Economic Assumptions	
**Waste plastics**	Available free of charge
**Natural gas (EUR /GJ)**	5
**Cooling water price EUR /ton**	0.01
**Waste disposal (EUR /t)**	10
**Plant construction time (year)**	3
**Plant life (years)**	30
**Maintenance**	3.5% of OPEX
**Discount rate**	0.08
**Administration**	30% Labor Cost
**Labor cost EUR /person**	45,000
**Offsite unit and utilities**	25% from equipment cost
**Stream factor**	0.95
**Daily number of shifts**	3
**Land and salvage (MMEUR)**	10% of FCI
**Working capital (MMEUR)**	10% of FCI
**Taxation rate (%)**	15
**Ratio of recycling methanol solvent**	0.01
**Price of methanol (EUR /ton)**	400
**Price of boiling water 2017 MEUR /ton**	2.03
**x**	0.60
**CEPCI (2021)**	620
